# The importance of being edgy: cell geometric edges as an emerging polar domain in plant cells

**DOI:** 10.1111/jmi.12847

**Published:** 2019-11-28

**Authors:** L. ELLIOTT, C. KIRCHHELLE

**Affiliations:** ^1^ Department of Plant Sciences University of Oxford South Parks Road Oxford UK

**Keywords:** Cell edges, cell geometry, cell polarity, CLASP, plant morphogenesis, RAB‐A5c, SOSEKI

## Abstract

Polarity is an essential feature of multicellular organisms and underpins growth and development as well as physiological functions. In polyhedral plant cells, polar domains at different faces have been studied in detail. In recent years, cell edges (where two faces meet) have emerged as discrete spatial domains with distinct biochemical identities. Here, we review and discuss recent advances in our understanding of cell edges as functional polar domains in plant cells and other organisms, highlighting conceptual parallels and open questions regarding edge polarity.

## Introduction

Polarity is a near‐universal feature in biology and exists across scales. For example, in higher plants, one of the first steps in embryo development is the establishment of the apical‐basal axis from which the plant's entire body plan is elaborated. At the organ level, additional polarity axes (e.g. proximal‐distal, adaxial–abaxial, outer–inner) are involved in establishing and maintaining plant organ shape and physiological function. This macroscopic polarity at the whole plant and organ level emerges from polarity at the cellular and subcellular level. For instance, the establishment of different organ shapes necessitates directional (polar) growth at the cellular level, which in turn depends on the establishment of biochemical and structural polarity within the cell and the cell wall. Advances in microscopy techniques over the last 20 years, particularly in the use of confocal laser‐scanning microscopy for live‐cell imaging, have revealed the polar distributions of many subcellular components (organelles as well as individual proteins) within plant cells, and have greatly advanced our understanding of how polarity at the cellular and subcellular scale translates into shape and function at the cell, tissue, and organ scales.

Depending on their developmental context, plant cells can adopt different shapes and polarities, which are often functionally linked. An extreme example are tip‐growing cells, which include root hairs and pollen tubes in angiosperms as well as caulonema and protonema cells in bryophytes. In these cells, growth is confined to a small region at the tip of an extending tubular structure, which is underpinned by a highly polarised intracellular machinery. In particular, Rho Of Plants (ROP) GTPases and their interactors have been characterised as significant molecular determinants of polarity in tip‐growing cells (Feiguelman *et al*., 2018; Denninger *et al*., 2019). For instance, tip‐localised ROPs have been implicated in organising a polarised actin cytoskeleton in root hairs, pollen tubes and protonema cells, which is in turn required for targeted transport of secretory trafficking towards the tip (Molendijk *et al*., 2001; Gu *et al*., 2003; Burkart *et al*., 2015). Another striking example for morphologically and biochemically polarised cells are trichomes of *Arabidopsis thaliana*, which specify two distinct apical and basal domains with distinct cell wall compositions and structures. This cell wall polarisation was functionally linked to the establishment of apical and basal domains with distinct lipid signatures in the plasma membrane, which in turn recruit different subunits of the exocyst complex, which is involved in targeted secretion (Kubátová *et al*., 2019). Cell polarity is also critical during the development of stomata in leaves, which involves a series of asymmetric cell divisions. Regulation of these asymmetric divisions depends on the polarised protein BREAKING OF ASYMMETRY IN THE STOMATAL LINEAGE (BASL), which localises to a crescent‐shaped subcellular domain in the cell periphery that predicts the location of the larger of the two future daughter cells (Dong *et al*., 2009; Mansfield *et al*., 2018). BASL acts alongside POLAR LOCALISATION DURING ASYMMETRIC DIVISION AND REDISTRIBUTION (POLAR), another asymmetrically localised protein that activates a MAPK‐based signalling cascade regulating the transcription factor SPEECHLESS (SPCH), which in turn drives asymmetric division in the stomata lineage (Pillitteri *et al*., 2011; Houbaert *et al*., 2018). Intriguingly, neither BASL or POLAR are membrane‐bound proteins, demonstrating that plant cells can set up polar domains within their cytoplasm.

These striking examples from specialised cell lineages illustrate the range of cellular responses involving some form of cell polarity: from cytoskeletal and endomembrane organisation to transcriptional regulation. For the remainder of this review however, we will focus on polyhedral, diffusely growing plant cells, which constitute the majority of cells in developing plant organs and in which polarity plays an equally essential role.

## Polarity in polyhedral cells

**Figure 1:**
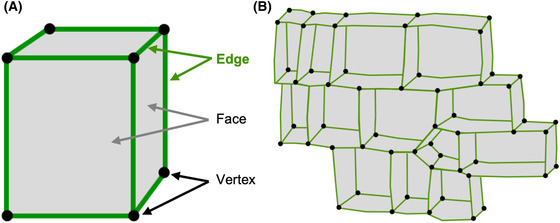
The cell edge as a geometric domain. Cartoon illustration geometric faces, edges and vertices on a single cell (A) and within a tissue context (B).

The often‐polyhedral shape of plant cells is one of their most striking features and has inspired a rich body of historical work employing mathematical rules to describe cell geometry and growth (Hofmeister, 1867; Sachs, 1877; Errera, 1888; Korn, 1982). In mathematical terms, cells can be described as geometric bodies with different faces (shared with neighbouring cells with the exception of the organ surface), edges (where two faces meet), and vertices (where three edges meet in a point; Fig. 1A). Experimental work in recent decades has revealed that these geometric domains can possess corresponding distinct biochemical identities through accumulation of polar proteins and cell components.

**Figure 2:**
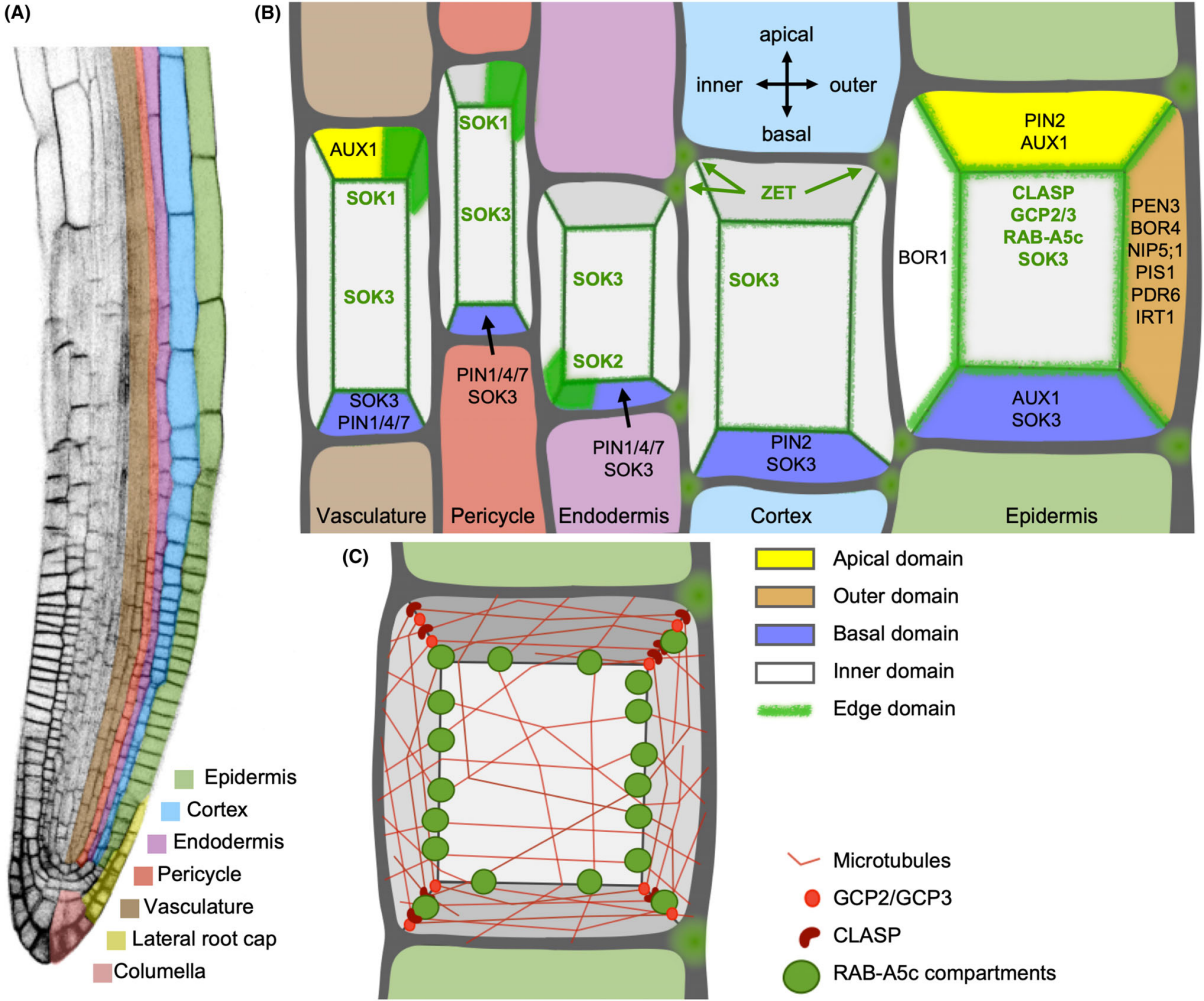
Polarity in the *Arabidopsis* root. (A) Tissue organisation in the *Arabidopsis* root. (B) Cartoon representation of different polar domains in different tissue layers of the *Arabidopsis* root. (C) Cartoon representation of edge‐localised proteins in *Arabidopsis* root epidermal cells.

Polar proteins at different faces have been studied in particular detail in the *Arabidopsis* root. In this system, cell faces are defined as apical, basal and lateral depending on their orientation relative to the root's main axis. Each of these faces can accumulate distinct proteins, resulting in unique identities and complex cell polarity. These proteins prominently include plasma‐membrane localised transporters of the phytohormone auxin, such as PIN‐FORMED (PIN) auxin efflux proteins and AUXIN RESISTANT1/LIKE AUXIN RESISTANT1 (AUX1/LAX) influx proteins. PIN and AUX1/LAX proteins mark the apical and basal domains of the plasma membrane, with the same PIN often labelling different domains in different cell types (Gälweiler *et al*., 1998; Müller *et al*., 1998; Swarup *et al*., 2001; Friml *et al*., 2002a, b) (Figs. 2A, B). The polar localisation of PIN and AUX1/LAX transporters is essential to establish and maintain tissue‐level auxin gradients, which are in turn pivotal to establish polarity axes at the tissue and organ scale during development (Friml *et al*., 2002a; Friml *et al*., 2003; Reinhardt *et al*., 2003; Swarup *et al*., 2004; Blilou *et al*., 2005; Swarup *et al*., 2008; Krecek *et al*., 2009; Ugartechea‐Chirino *et al*., 2010), reviewed by Robert *et al*. (2015), Zhou & Luo (2018). Outer and inner lateral domains of the *Arabidopsis* root epidermis are also defined by a variety of plasma membrane‐localised proteins. In contrast to the pivotal role of auxin transporters in establishing tissue‐level polarity during growth, these laterally polarised proteins have largely been associated with nutrient and water transport [REQUIRES HIGH BORON4 (BOR4), NOD26‐LIKE INTRINSIC PROTEIN 5;1 (NIP5;1), REQUIRES HIGH BORON1 (BOR1), IRON‐REGULATED TRANSPORTER1 (IRT1)] (Miwa *et al*., 2007; Takano *et al*., 2010; Barberon *et al*., 2014) or pathogen defence [PENETRATION3 (PEN3), POLAR AUXIN TRANSPORT INHIBITOR SENSITIVE1 (PIS1), PLEIOTROPIC DRUG RESISTANCE6 (PDR6)] (Strader & Bartel, 2009; Ruzicka *et al*., 2010; Khare *et al*., 2017) as well as trafficking regulators of these polarised proteins (Fendrych *et al*., 2013; reviewed by Nakamura & Grebe, 2018) (Fig. 2B).

Polarity does not stop at the plasma membrane: establishing polarity within the cell wall surrounding each cell is an essential aspect of plant growth. In addition to the well‐described oriented deposition of cellulose within the cell wall which provides structural anisotropy (Green, 1962), plants can also establish distinct cell wall polarities at different faces. In the *Arabidopsis* hypocotyl for example, cellulose microfibril organisation is highly anisotropic at the inner lateral face but significantly more isotropic at the outer lateral face, which in turn depends on differences in cortical microtubule arrangement at these faces (Crowell *et al*., 2011). Furthermore, selective modification of cell wall matrix properties at different faces has also been implicated in the establishment of anisotropic growth in the *Arabidopsis* hypocotyl (Peaucelle *et al*., 2015). In hypocotyl epidermis cells, de‐methyesterification of pectin at longitudinal faces through polarised pectin methylesterase (PME) activity has been linked to wall loosening and consequently, increased cell wall extensibility (Peaucelle *et al*., 2015). Polarised modification of cell walls at specific faces has also been described in other contexts: for example, lignin is deposited in a highly polarised fashion in endocarp *b* cells in ripening fruit pods of *Cardamine hirsuta*, a feature associated with the explosive dispersal of *Cardamine* seeds (Vaughn *et al*., 2011; Hofhuis *et al*., 2016).

Although the importance of facial polarity in *Arabidopsis* cells and cell walls has been recognised for some time, in recent years it has become apparent that plant cells additionally specify another geometric domain within polyhedral cells: their geometric edges.

## The cytoskeleton at cell geometric edges

Some of first descriptions of plant cell geometric edges as a distinct biochemical domain come from historic observations of the cytoskeleton at these regions. In root cells of the water fern *Azolla*, cell edges are densely populated with microtubules and were proposed to act as domains of microtubule nucleation (Gunning *et al*., 1978; Gunning, 1980). Similar observations were also made in leaflet cells of the fern *Adiantum capillus‐veneris* (Panteris *et al*., 1993a, b). Similarly, the observation that cell edges are enriched in preprophase microtubule bands compared to the cell faces in the liverwort *Marchantia paleacea* and the fern *Adiantum capillus‐veneris* inspired the proposal that edges act as microtubule organisers (Panteris *et al*., 1991; Apostolakos & Galatis, 1992). More recent work in *Arabidopsis* has provided insights into the molecular mechanisms underlying these early observations (Fig. 2C). The previously hypothesised role for cell edges in microtubule nucleation is supported by the observation that components of the microtubule‐nucleating γ‐tubulin complex, GAMMA TUBULIN COMPLEX PROTEINS2 and 3 (GCP2 and GCP3), are enriched at geometric edges in *Arabidopsis* root cells (Ambrose & Wasteneys, 2011). Furthermore, the microtubule‐associated CLIP‐ASSOCIATED PROTEIN (CLASP) localises to sharp geometric edges of growing cells in the root and shoot of *Arabidopsis* where it contributes to organising the cortical microtubule array (Ambrose *et al*., 2011; Le & Ambrose, 2018). Cortical microtubules encountering sharp edges typically undergo catastrophe (rapid depolymerisation at the plus end). The presence of CLASP at edges can prevent microtubule catastrophe, thus allowing cortical microtubules to cross sharp edges (Ambrose *et al*., 2011). It has been observed in computational models of self‐organising microtubule arrays on realistic cell geometries that cell and edge geometry has an important effect on cortical microtubule organisation at the cellular scale (Chakrabortty *et al*., 2018a, b). Microtubule nucleation and CLASP‐mediated crossing at cell edges constitute two different regulatory mechanisms beyond purely geometric cues through which cells can control cell‐wide microtubule organisation. Importantly, if different edges within a cell have different microtubule organising activities, such edges can provide vectorial information to organise directional microtubule arrays at faces. Indeed, neither CLASP nor GCP2/3 are evenly distributed along all edges. In primary root tips, GCP2/3‐GFP strongly label transverse cell edges (perpendicular to growth direction) but are virtually absent from longitudinal edges (parallel to growth direction) (Ambrose & Wasteneys, 2011). In lateral roots by contrast, GCP2‐GFP localises to both longitudinal and transverse edges, although it is approximately two‐fold enriched at transverse edges (Kirchhelle *et al*., 2019). CLASP is preferentially enriched at the sharp transverse edges of recently divided root meristematic cells, but enriched at longitudinal edges in elongation zone cells (Ambrose *et al*., 2011). This indicates that both CLASP and GCP2/3 activity promote the presence of microtubules in longitudinal orientation in meristematic cells, matching the observation that in the absence of CLASP, microtubule arrays in meristematic cells become hyperaligned in transverse orientation (Ambrose *et al*., 2011). These observed differences in CLASP and GCP2/3 localisation may simply reflect different edge geometries. However, it is also possible that other factors are involved in preferential recruitment of CLASP and GCP2/3 to specific edges, implying different edges may have different identities analogous to the complex polarity at different cell faces in the root. What is the biological significance of organising cortical microtubule arrays at edges? Cortical microtubule array orientation controls the organisation of cellulose microfibrils through guidance of Cellulose Synthase Complexes, and consequently, directional growth. Mutants in both CLASP and GCP‐components have severe growth defects (Ambrose *et al*., 2007; Nakamura & Hashimoto, 2009); however, both proteins also have edge‐independent functions which likely dominate the observed phenotypes. Without strategies specifically abolishing edge‐localisation or ‐activity of CLASP and GCP2/3, it is difficult to experimentally assess the phenotypic consequences of disrupting edge‐based microtubule organisers.

Considering the emerging pivotal role of cell edges in organising microtubules in polyhedral cells of growing organs, the question arises whether cell edges play a similar role in organising the actin cytoskeleton. So far, comparatively little attention has been paid to the pattern of the actin cytoskeleton in such cells and it is therefore accordingly uncertain whether cell edges may also be sites of actin nucleation or organisation. However, some *in planta* imaging of fluorophore‐tagged actin‐binding fimbrin and fimbrin‐truncations in roots, lateral roots and hypocotyls of *Arabidopsis* indicates strong enrichment of actin at the cell periphery and possibly at cell geometric edges (Wang *et al*., 2004; Voigt *et al*., 2005; Wang *et al*., 2008). Furthermore, actin‐associated proteins have also been reported to localise to the cell periphery, with some labelling cell vertices: components of the membrane‐localised SCAR complex, which activates the conserved actin‐nucleating APR2/3 complex, localise to cell vertices in both root and shoot tissue (Dyachok *et al*., 2008; Dyachok *et al*., 2011). It is worth noting however that these localisation patterns may also represent polar localisation to anticlinal cell edges and should perhaps be re‐examined in the current context of greater appreciation of cell geometric edges as polar domains and sites of cytoskeleton initiation.

## Membrane‐associated proteins at cell edges

In their seminal electron microscopy studies of *Azolla*, Gunning and colleagues noted microtubules at plant cell edges often coincided with vesicles (Gunning *et al*., 1978), and similar observations were made in leaflet cells of *Adiantum capillus‐veneris* (Panteris *et al*., 1993a, b). Recent work has revealed that such vesicles at cell edges can have biochemically distinct identities: in lateral organ primordia and young primary roots of *Arabidopsis*, vesicular compartments labelled by the small GTPase RAB‐A5c accumulate specifically at cell edges (Kirchhelle *et al*., 2016; Kirchhelle *et al*., 2019) (Fig. 2C). Localisation of RAB‐A5c mutant variants and the endocytic tracer dye FM4‐64 indicates that these cell edge compartments derive from the post‐Golgi sorting hub, the *trans*‐Golgi network/early endosome (TGN/EE), and eventually fuse with the plasma membrane, indicating that cells specify an exocytic trafficking route specifically to their geometric edges (Kirchhelle *et al*., 2016). Inhibition of RAB‐A5c function through conditional expression of a dominant‐inhibitory Rab mutant variant results in a shift from anisotropic (directional) to near‐isotropic growth in lateral root epidermal cells (Kirchhelle *et al*., 2016). This suggests that a RAB‐A5c‐mediated edge‐directed trafficking route is required for directional growth in *Arabidopsis*. Interestingly, this effect on directional growth is likely independent of the role cell edges play in organising cortical microtubule arrays: RAB‐A5c and CLASP or GCP2 localise to cell edges independently of each other, and there is little overlap between RAB‐A5c and CLASP or GCP2 at cell edges (Kirchhelle *et al*., 2016; Kirchhelle *et al*., 2019). Surprisingly, cortical microtubules were significantly more aligned in transverse orientation when RAB‐A5c function was inhibited, an orientation typically associated with anisotropic rather than the observed near‐isotropic growth. When microtubule reorganisation was perturbed genetically or pharmacologically concomitant with RAB‐A5c inhibition, the growth defects were synergistically enhanced (Kirchhelle *et al*., 2019). This indicates the observed microtubule reorganisation was a compensatory response, and that exocytic trafficking to cell edges affects directional growth through a different mechanism. Like CLASP and GCP2, RAB‐A5c‐labelled cell edge compartments do not associate uniformly with all cell edges in growing tissues, further supporting the notion that different edges may have different polarities. In contrast to CLASP and GCP2 however, RAB‐A5c is significantly enriched at longitudinal cell edges in meristematic lateral root cells (Kirchhelle *et al*., 2019). Similar to CLASP, RAB‐A5c is largely confined to longitudinal edges in elongation zone cells (Kirchhelle *et al*., 2016). Although neither CLASP nor GCP2 appears to be necessary for RAB‐A5c edge‐localisation *per se*, the relative distribution of RAB‐A5c across different cell edge is sensitive to mutations in these proteins. The *clasp‐1* background significantly enhances localisation of RAB‐A5c to longitudinal edges of meristematic cells, whereas the GCP2 mutant *spr3* results in increased RAB‐A5c localisation to transverse cell edges (Kirchhelle *et al*., 2019). As RAB‐A5c localisation at cell edges is dependent on microtubules (Kirchhelle *et al*., 2016), these differences in relative RAB‐A5c enrichment are likely indirect and caused by changes in cell‐wide microtubule organisation in these mutants. However, they highlight a possible mode of action for cross‐talk between different proteins localising to cell edge domains through convergence on microtubule arrays.

In addition to the discovery of discrete edge‐localised membrane compartments, the significance of cell edges as a discrete polar domain has been highlighted by the recent identification of the SOSEKI (SOK) family of proteins in *Arabidopsis* (Yoshida *et al*., 2019). The five SOK proteins appear to peripherally associate with the plasma membrane and occupy a subcellular domain including cell edges and part of the adjacent cell faces. Different SOK members identify different subcellular domains: for instance, SOK1:YFP localises to the apical outer edge in young vascular cells, whereas SOK2:YFP localises to basal cell edges in root endodermal cells (Yoshida *et al*., 2019) (Fig. 2B). Mis‐expression of SOK1 induced abnormal cell division orientations in roots, but the function of the remaining SOK proteins remains unknown. SOK proteins are expressed in the embryo as well as primary and lateral roots and intriguingly, localise to the same subcellular polar domain relative to the main organ axes in both the embryo and the root (Yoshida *et al*., 2019). This observation gives rise to the attractive hypothesis that SOK proteins may integrate information from the apical‐basal and outer–inner polar axes. Unlike cell‐edge localised RAB‐A5c, SOK edge localisation is not sensitive to pharmacological disruption of either microtubules or actin (Yoshida *et al*., 2019), indicating other mechanisms are involved in establishing their polarity. Intriguingly, SOK proteins contain a DUF966 domain, which has structural similarities to the DIX domain found in known regulators of metazoan cell polarity. Polar localisation of SOK1 is lost in a protein deletion lacking the DUF966 domain and its adjacent regions. Thus, specific edge‐polarity of SOK proteins has been associated with a protein region with similarity to metazoan polarity determinants, providing the first polarity determinant of an edge‐localised protein in plants.

## Edge polarity in the plant cell wall

Cell edges have also emerged as a domain of interest in the context of cell wall biology. In particular, it has been argued that the high, undirected turgor pressure that promotes a spherical cell shape creates significant mechanical stresses at the edges of polyhedral cells (Jarvis *et al*., 2003), which have to be counteracted by strengthening of the cell wall specifically at edges. There is a rich body of literature investigating the composition of the cell wall in differentiated tissues, revealing different mechanisms through which such mechanical strengthening may be accomplished. For instance, cell edges at intercellular spaces in parenchymous tissue of various tubers, roots and fruit are rich in pectin, particularly in its de‐methylesterified form (Bush *et al*., 2001; Parker *et al*., 2001; Jarvis *et al*., 2003; Guillemin *et al*., 2005; Szymańska‐Chargot *et al*., 2016). De‐methylesterified pectin can mechanically stiffen the cell wall through calcium cross‐linking (Wolf & Greiner, 2012), and indeed calcium itself can be enriched in the cell wall around cell vertices (Guglielmino *et al*., 1997). Furthermore, cell edges in some species are additionally enriched in phenolic compounds involved in cell‐cell adhesion (Parr *et al*., 1996; Waldron *et al*., 1997; Parker *et al*., 2003; Suslov *et al*., 2009). For example, cell edges of Chinese water chestnut were found to be strongly enriched in ferulic acid (FA) (Parr *et al*., 1996). This has been proposed to locally strengthen the cell wall through increased oxidative cross‐linking of FA‐esterified pectic polysaccharides, which become less soluble in water as a result (Waldron *et al*., 1997; Parker *et al*., 2003). In addition to cell edges at intracellular spaces, cell edges at the outer faces of onion epidermal cells are different in composition to adjacent cell faces as they have a lower cellulose content (Suslov *et al*., 2009). These edges were found to be softer than faces in indentation experiments (Routier‐Kierzkowska *et al*., 2012), but surprisingly, also appeared to be stronger than cell faces under tensile loading (Zamil *et al*., 2013; Zamil *et al*., 2014). Other reports also describe edge‐enrichment of structurally supportive lignin and lignin‐related enzymes in some tissues and species (Tirumalai *et al*., 1996; Agarwal, 2006; Zeng *et al*., 2017; Yi Chou *et al*., 2018). Lignin enrichment is also known to occur along cell edges in some specialised *Arabidopsis* cell types. The formation of a lignin ‘brace’ delineating cell edges around cells in abscission zones of *Arabidopsis* floral organs is required for proper floral abscission and may function by restricting diffusion of cell wall digesting enzymes, ensuring their concentration around the abscission site (Lee *et al*., 2018).

Less is known about the importance of local cell wall modifications in the context of growing tissues. In such tissues, cell edges are expected to accumulate mechanical stress not just for geometrical reasons as outlined above. Cell edges also connect faces on the same cell or neighbouring cells that can grow at significantly different rates and in different directions, which may lead to a build‐up of shear stresses at the edge domain. This implies a possible need for selective stiffening through localised accumulation or modification of cell wall carbohydrates, which in turn necessitates targeted intracellular transport of either cell wall carbohydrates or their associated biosynthetic machinery. The recently described putatively secretory transport pathway to cell edges mediated by RAB‐A5c is a prime candidate for such an activity. Supporting this notion, 3D Finite Element models predict that local reduction of cell wall stiffness at edges can lead to cell swelling resembling that observed in plants in which RAB‐A5c‐mediated trafficking was inhibited (Kirchhelle *et al*., 2019). When RAB‐A5c function was inhibited, cells lost their ability for directional growth. This may be a simple consequence of outward bulging, but considering the non‐uniform distribution of RAB‐A5c compartments at different cell edges, it is also possible that selective modification of cell wall properties at different edges within a cell can dictate a preferential growth direction. Intriguingly, a similar prediction was made in an early conceptual model of plant growth which considered plant cell edges, faces and vertices as distinct elements (Korn, 1982). This model concludes that the rate of cell edge growth was primary and causal in determining the growth of the model epidermis.

Additional evidence that cell edge polarity is important during growth comes from studies of the apoplast‐localised predicted β‐1,3 glucanase ZERZAUST (ZET) (Fulton *et al*., 2009; Vaddepalli *et al*., 2017) (Fig. 2B). In cortical cells of *Arabidopsis* roots, ZET is confined to longitudinal edges, where it associates with the cell wall. The precise function of ZET at cell edges is unclear but loss of ZET is associated with defects in trichoblast specification and morphological abnormalities including stunted growth and organ twisting in aerial organs (Fulton *et al*., 2009). Intriguingly, Fourier‐transformed infrared‐spectroscopy (FTIR) of *zet* mutant cell wall preparations revealed alterations in cell wall composition (Vaddepalli *et al*., 2017). It is not yet clear whether these alterations are a direct or indirect consequence of loss of ZET function, whether they reflect global changes in cell wall composition or local changes at the edge domain, and how they are functionally linked to the observed morphological phenotypes.

In addition to this emerging role in growth control of cell edges, it is interesting to note that localisation of *Arabidopsis* SOK proteins to the cell edge periphery is dependent on cell wall integrity (Yoshida *et al*., 2019), indicating feedback between the cell wall and intracellular polarity. Whether this dependence represents a direct role for the cell wall in maintenance of cell geometric edges as a discrete cellular domain, or is due to disruption of cell geometry or cell mechanical properties by cell‐wall disrupting treatments remains unclear.

## Cell edges – plants and beyond

Having long featured in mathematical descriptions of plant cells, cell edges have recently also emerged as biochemically distinct, polar domains within cells. Cell edge polarity has important functions during growth, yet many questions regarding the definition and function of cell geometric edges as discrete cellular domains remain open. For instance, the differences between RAB‐A5c and SOK subcellular localisation, dynamics and (possibly) function suggest that these represent separate edge‐polarised systems in plant cells. How is edge polarity of these systems established and maintained? What roles do these systems respectively fulfil, and how are they related to each other? Answering these questions will depend on the identification and characterisation of further molecular components in each system.

Emerging knowledge about the cell edge domain in plants also raises questions regarding an analogous role of cell edges as discrete cellular domains in other multicellular organisms. Polyhedral cells with sharply defined edges also exist within metazoan tissues, particularly within epithelia. Within epithelial sheets, tricellular junctions (the edges where three cells meet) accumulate distinct proteins which form tight junctions to maintain the epithelium's barrier function (Staehelin, 1973; Ikenouchi *et al*., 2005; Byri *et al*., 2015). Tricellular junctions are also believed to be major tension‐bearing elements in epithelial sheets (Oda *et al*., 2014). Recently, an intriguing additional function for tricellular junctions during *Drosophila* embryo development has been identified. Tricellular junctions accumulated the dynein‐associated protein Mud, which in association with microtubules act as force‐generators to orient the mitotic spindle and thus cell division plane orientation (Bosveld *et al*., 2016). It has also been reported that pancreatic β cells (which do not exhibit pronounced basal‐apical polarity) can exhibit edge polarity, as geometric edges in these cells were enriched in proteins involved in cell‐cell adhesion, signalling and secretion (Geron *et al*., 2015). This edge‐enrichment was not observed in other studies though and it has been suggested that such differences may arise from use of cultured islets versus islet slices (Low *et al*., 2014; Gan et al., 2017). Although pancreatic β cells are principally capable of edge polarisation, it therefore remains unclear whether this polarity is physiological significant in real tissues.

Little is known about a role of cell edges as polar domains in other multicellular organisms, although it should be noted recent work revealed edge‐enrichment of certain fungal cell‐wall associated glucan elongations proteins (GELs) in spores of *Magnaporthe oryzae* (rice blast fungus) (Samalova *et al*., 2017). GEL proteins likely assist with modification of the fungal cell wall during growth of infection structures and in the early‐developing spores of *Magnaporthe oryzae*, although the functional role of their edge‐specific enrichment remains unknown.

Although different molecular mechanisms are involved and knowledge about cell edge polarity is still sparse, there are some interesting conceptual parallels between the observed role of cell edges in different organisms: namely, cell edges can be polarised with respect to membrane trafficking and cytoskeletal organisation, and they can be involved in maintaining cell geometry and division plane orientation (possibly through using cell edges as landmarks), and they may be tension‐ or load‐bearing points, necessitating local modification of the extracellular matrix. Are edges universally important polar domains? Our understanding of the mechanisms underpinning cell edge polarity and its diverse functions is still in its infancy, but these observations provide an exciting starting point to further explore this question.

## References

[jmi12847-bib-0001] Agarwal, U.P. (2006) Raman imaging to investigate ultrastructure and composition of plant cell walls: distribution of lignin and cellulose in black spruce wood (*Picea mariana*). Planta 224, 1141–1153. 16761135 10.1007/s00425-006-0295-z

[jmi12847-bib-0002] Ambrose, C. , Allard, J.F. , Cytrynbaum, E.N. & Wasteneys, G.O. (2011) A CLASP‐modulated cell edge barrier mechanism drives cell‐wide cortical microtubule organization in *Arabidopsis* . Nat. Commun. 2, 430. 21847104 10.1038/ncomms1444 PMC3265373

[jmi12847-bib-0003] Ambrose, C. & Wasteneys, G.O. (2011) Cell edges accumulate Gamma tubulin complex components and nucleate microtubules following cytokinesis in *Arabidopsis thaliana* . PLOS ONE 6, e27423. 22110647 10.1371/journal.pone.0027423 PMC3212562

[jmi12847-bib-0004] Ambrose, J.C. , Shoji, T. , Kotzer, A.M. , Pighin, J.A. & Wasteneys, G.O. (2007) The *Arabidopsis* CLASP gene encodes a microtubule‐associated protein involved in cell expansion and division. Plant Cell 19, 2763–2775. 17873093 10.1105/tpc.107.053777 PMC2048705

[jmi12847-bib-0005] Apostolakos, P. & Galatis, B. (1992) Patterns of microtubule organization in two polyhedral cell types in the gametophyte of the liverwort *Marchantia paleacea* Bert. New Phytol. 122, 165–178. 10.1111/j.1469-8137.1992.tb00063.x 33874052

[jmi12847-bib-0006] Barberon, M. , Dubeaux, G. , Kolb, C. , Isono, E. , Zelazny, E. & Vert, G. (2014) Polarization of iron‐regulated transporter 1 (IRT1) to the plant‐soil interface plays crucial role in metal homeostasis. Proc. Nat. Acad. Sci. U.S.A. 111, 8293–8298. 10.1073/pnas.1402262111 PMC4050562 24843126

[jmi12847-bib-0007] Blilou, I. , Xu, J. , Wildwater, M. *et al* (2005) The PIN auxin efflux facilitator network controls growth and patterning in *Arabidopsis* roots. Nature 433, 39–44. 15635403 10.1038/nature03184

[jmi12847-bib-0008] Bosveld, F. , Markova, O. , Guirao, B. *et al* (2016) Epithelial tricellular junctions act as interphase cell shape sensors to orient mitosis. Nature 530, 495–498. 26886796 10.1038/nature16970 PMC5450930

[jmi12847-bib-0009] Burkart, G.M. , Baskin, T.I. & Bezanilla, M. (2015) A family of ROP proteins that suppresses actin dynamics, and is essential for polarized growth and cell adhesion. J. Cell Sci. 128, 2553–2564. 26045445 10.1242/jcs.172445

[jmi12847-bib-0010] Bush, M.S. , Marry, M. , Huxham, M.I. , Jarvis, M.C. & McCann, M.C. (2001) Developmental regulation of pectic epitopes during potato tuberisation. Planta 213, 869–880. 11722123 10.1007/s004250100570

[jmi12847-bib-0011] Byri, S. , Misra, T. , Syed, Z.A. *et al* (2015) The triple‐repeat protein Anakonda controls Epithelial tricellular junction formation in Drosophila. Dev. Cell 33, 535–548. 25982676 10.1016/j.devcel.2015.03.023

[jmi12847-bib-0012] Chakrabortty, B. , Blilou, I. , Scheres, B. & Mulder, B.M. (2018a) A computational framework for cortical microtubule dynamics in realistically shaped plant cells. PLOS Comput. Biol. 14, e1005959. 29394250 10.1371/journal.pcbi.1005959 PMC5812663

[jmi12847-bib-0013] Chakrabortty, B. , Willemsen, V. , de Zeeuw, T. , Liao, C.‐Y. , Weijers, D. , Mulder, B. & Scheres, B. (2018b) A plausible microtubule‐based mechanism for cell division orientation in plant embryogenesis. Curr. Biol. 28, 3031–3043. 30245102 10.1016/j.cub.2018.07.025

[jmi12847-bib-0014] Crowell, E.F. , Timpano, H. , Desprez, T. , Franssen‐Verheijen, T. , Emons, A.‐M. , Höfte, H. & Vernhettes, S. (2011) Differential regulation of cellulose orientation at the inner and outer face of epidermal cells in the *Arabidopsis* hypocotyl. Plant Cell 23, 2592–2605. 21742992 10.1105/tpc.111.087338 PMC3226210

[jmi12847-bib-0015] Denninger, P. , Reichelt, A. , Schmidt, V.A.F. *et al* (2019) Distinct RopGEFs successively drive polarization and outgrowth of root hairs. Curr. Biol. 29, 1854–1865. 31104938 10.1016/j.cub.2019.04.059

[jmi12847-bib-0016] Dong, J. , MacAlister, C.A. & Bergmann, D.C. (2009). BASL controls asymmetric cell division in *Arabidopsis* . Cell 137, 1320–1330. 19523675 10.1016/j.cell.2009.04.018 PMC4105981

[jmi12847-bib-0017] Dyachok, J. , Shao, M.‐R. , Vaughn, K. , Bowling, A. , Facette, M. , Djakovic, S. , Clark, L. & Smith, L. (2008) Plasma membrane‐associated SCAR complex subunits promote cortical F‐actin accumulation and normal growth characteristics in *Arabidopsis* roots. Mol. Plant 1, 990–1006. 19825598 10.1093/mp/ssn059

[jmi12847-bib-0018] Dyachok, J. , Zhu, L. , Liao, F. , He, J. , Huq, E. & Blancaflor, E.B. (2011) SCAR mediates light‐induced root elongation in *Arabidopsis* through photoreceptors and proteasomes. Plant Cell 23, 3610–3626. 21972261 10.1105/tpc.111.088823 PMC3229138

[jmi12847-bib-0019] Errera, L. (1888) Über Zellformen und Seifenblasen. Botanisches Centralblatt 34, 395–399.

[jmi12847-bib-0020] Feiguelman, G. , Fu, Y. & Yalovsky, S. (2018) ROP GTPases structure‐function and signaling pathways. Plant Physiol. 176, 57–79. 29150557 10.1104/pp.17.01415 PMC5761820

[jmi12847-bib-0021] Fendrych, M. , Synek, L. , Pečenková, T. , Drdová, E.J. , Sekereš, J. , de Rycke, R. , Nowack, M.K. & Žárský, V. (2013) Visualization of the exocyst complex dynamics at the plasma membrane of *Arabidopsis thaliana* . Mol. Biol. Cell 24, 510–520. 23283982 10.1091/mbc.E12-06-0492 PMC3571873

[jmi12847-bib-0022] Friml, J. , Benková, E. , Blilou, I. *et al* (2002a) AtPIN4 mediates sink‐driven auxin gradients and root patterning in *Arabidopsis* . Cell 108, 661–673. 11893337 10.1016/s0092-8674(02)00656-6

[jmi12847-bib-0023] Friml, J. , Vieten, A. , Sauer, M. , Weijers, D. , Schwarz, H. , Hamann, T. , Offringa, R. & Jürgens, G. (2003) Efflux‐dependent auxin gradients establish the apical‐basal axis of *Arabidopsis* . Nature 426, 147–153. 14614497 10.1038/nature02085

[jmi12847-bib-0024] Friml, J. , Wiśniewska, J. , Benková, E. , Mendgen, K. & Palme, K. (2002b) Lateral relocation of auxin efflux regulator PIN3 mediates tropism in *Arabidopsis* . Nature 415, 806–809. 11845211 10.1038/415806a

[jmi12847-bib-0025] Fulton, L. , Batoux, M. , Vaddepalli, P. *et al* (2009) DETORQUEO, QUIRKY, and ZERZAUST represent novel components involved in organ development mediated by the receptor‐like kinase STRUBBELIG in *Arabidopsis thaliana* . PLOS Genet. 5, e1000355. 19180193 10.1371/journal.pgen.1000355 PMC2628281

[jmi12847-bib-0026] Gälweiler, L. , Guan, C. , Müller, A. , Wisman, E. , Mendgen, K. , Yephremov, A. & Palme, K. (1998) Regulation of polar auxin transport by AtPIN1 in *Arabidopsis* vascular tissue. Science 282, 2226–2230. 9856939 10.1126/science.282.5397.2226

[jmi12847-bib-0027] Gan, W.J. , Zavortink, M. , Ludick, C. *et al* (2017) Cell polarity defines three distinct domains in pancreatic β‐cells. J. Cell Sci. 130, 143–151. 26919978 10.1242/jcs.185116 PMC5394774

[jmi12847-bib-0028] Geron, E. , Boura‐Halfon, S. , Schejter, E.D. & Shilo, B.‐Z. (2015) The edges of pancreatic Islet β cells constitute adhesive and signaling microdomains. Cell Reports 10, 317–325. 25600867 10.1016/j.celrep.2014.12.031

[jmi12847-bib-0029] Green, P.B. (1962) Mechanism for plant cellular morphogenesis. Science 138, 1404–1405. 17753861 10.1126/science.138.3548.1404

[jmi12847-bib-0030] Gu, Y. , Vernoud, V. , Fu, Y. & Yang, Z. (2003) ROP GTPase regulation of pollen tube growth through the dynamics of tip‐localized F‐actin. J. Exp. Bot. 54, 93–101. 12456759 10.1093/jxb/erg035

[jmi12847-bib-0031] Guglielmino, N. , Liberman, M. , Jauneau, A. , Vian, B. , Catesson, A.M. & Goldberg, R. (1997) Pectin immunolocalization and calcium visualization in differentiating derivatives from poplar cambium. Protoplasma 199, 151–160.

[jmi12847-bib-0032] Guillemin, F. , Guillon, F. , Bonnin, E. , Devaux, M.‐F. , Chevalier, T. , Knox, J.P. , Liners, F. & Thibault, J.‐F. (2005) Distribution of pectic epitopes in cell walls of the sugar beet root. Planta 222, 355–371. 15887026 10.1007/s00425-005-1535-3

[jmi12847-bib-0033] Gunning, B.E.S. , Hardham, A.R. & Hughes, J.E. (1978) Pre‐prophase bands of microtubules in all categories of formative and proliferative cell division in *Azolla* roots. Planta 143, 145–160. 24408366 10.1007/BF00387787

[jmi12847-bib-0034] Gunning, B.S. (1980) Spatial and temporal regulation of nucleating sites for arrays of cortical microtubules in root tip cells of the water fern *Azolla pinnata* . Euro. J. Cell Biol. 23, 53–65. 7460968

[jmi12847-bib-0035] Hofhuis, H. , Moulton, D. , Lessinnes, T. *et al* (2016) Morphomechanical innovation drives explosive seed dispersal. Cell 166, 222–233. 27264605 10.1016/j.cell.2016.05.002 PMC4930488

[jmi12847-bib-0036] Hofmeister, W. (1867) Die Lehre von der Pflanzenzelle. W. Engelmann, Leipzig, Germany.

[jmi12847-bib-0037] Houbaert, A. , Zhang, C. , Tiwari, M. *et al* (2018) POLAR‐guided signalling complex assembly and localization drive asymmetric cell division. Nature 563, 574. 30429609 10.1038/s41586-018-0714-x

[jmi12847-bib-0038] Ikenouchi, J. , Furuse, M. , Furuse, K. , Sasaki, H. , Tsukita, S. & Tsukita, S. (2005) Tricellulin constitutes a novel barrier at tricellular contacts of epithelial cells. J. Cell Biol. 171, 939–945. 16365161 10.1083/jcb.200510043 PMC2171318

[jmi12847-bib-0039] Jarvis, M.C. , Briggs, S.P.H. & Knox, J.P. (2003) Intercellular adhesion and cell separation in plants. Plant, Cell Environ. 26, 977–989.

[jmi12847-bib-0040] Khare, D. , Choi, H. , Huh, S.U. *et al* (2017) *Arabidopsis* ABCG34 contributes to defense against necrotrophic pathogens by mediating the secretion of camalexin. Proc. Nat. Acad. Sci. U.S.A. 114, E5712–E5720. 10.1073/pnas.1702259114 PMC5514727 28652324

[jmi12847-bib-0041] Kirchhelle, C. , Chow, C.‐M. , Foucart, C. *et al* (2016) The specification of geometric edges by a plant Rab GTPase is an essential cell‐patterning principle during organogenesis in *Arabidopsis* . Dev. Cell 36, 386–400. 26906735 10.1016/j.devcel.2016.01.020 PMC4766369

[jmi12847-bib-0042] Kirchhelle, C. , Garcia‐Gonzalez, D. , Irani, N.G. , Jérusalem, A. & Moore, I. (2019) Two mechanisms regulate directional cell growth in *Arabidopsis* lateral roots. Elife 8, e47988. 31355749 10.7554/eLife.47988 PMC6748828

[jmi12847-bib-0043] Korn, R.W. (1982) Positional specificity within plant cells. J. Theor. Biol. 95, 543–568.

[jmi12847-bib-0044] Krecek, P. , Skupa, P. , Libus, J. , Naramoto, S. , Tejos, R. , Friml, J. & Zazímalová, E. (2009) The PIN‐FORMED (PIN) protein family of auxin transporters. Genome Biol. 10, 249. 20053306 10.1186/gb-2009-10-12-249 PMC2812941

[jmi12847-bib-0045] Kubátová, Z. , Pejchar, P. , Potocký, M. , Sekereš, J. , Žárský, V. & Kulich, I. (2019) *Arabidopsis* trichome contains two plasma membrane domains with different lipid compositions which attract distinct EXO70 subunits. Int. J. Mol. Sci. 20, 3803. 10.3390/ijms20153803 PMC6695903 31382643

[jmi12847-bib-0046] Le, P.Y. & Ambrose, C. (2018) CLASP promotes stable tethering of endoplasmic microtubules to the cell cortex to maintain cytoplasmic stability in *Arabidopsis* meristematic cells. PLOS ONE 13, e0198521. 29894477 10.1371/journal.pone.0198521 PMC5997327

[jmi12847-bib-0047] Lee, Y. , Yoon, T.H. , Lee, J. *et al* (2018) A Lignin molecular brace controls precision processing of cell walls critical for surface integrity in *Arabidopsis* . Cell 173, 1468–1480.e9. 29731167 10.1016/j.cell.2018.03.060

[jmi12847-bib-0048] Low, J.T. , Zavortink, M. , Mitchell, J.M. , Gan, W.J. , Do, O.H. , Schwiening, C.J. , Gaisano, H.Y. & Thorn, P. (2014) Insulin secretion from beta cells in intact mouse islets is targeted towards the vasculature. Diabetologia 57, 1655–1663. 24795086 10.1007/s00125-014-3252-6 PMC4079948

[jmi12847-bib-0049] Luo, J. , Zhou, J.‐J. & Zhang, J.‐Z. (2018) Aux/IAA gene family in plants: molecular structure, regulation, and function. Int. J. Mol. Sci. 19, 259. 10.3390/ijms19010259 PMC5796205 29337875

[jmi12847-bib-0050] Mansfield, C. , Newman, J.L. , Olsson, T.S.G. , Hartley, M. , Chan, J. & Coen, E. 2018 Ectopic BASL reveals tissue cell polarity throughout leaf development in *Arabidopsis thaliana* . Curr. Biol. 28, 2638–2646.e4. 30100337 10.1016/j.cub.2018.06.019 PMC6109230

[jmi12847-bib-0051] Miwa, K. , Takano, J. , Omori, H. , Seki, M. , Shinozaki, K. & Fujiwara, T. (2007) Plants tolerant of high boron levels. Science 318, 1417. 18048682 10.1126/science.1146634

[jmi12847-bib-0052] Molendijk, A.J. , Bischoff, F. , Rajendrakumar, C.S.V. , Friml, J. , Braun, M. , Gilroy, S. & Palme, K. (2001) *Arabidopsis thaliana* Rop GTPases are localized to tips of root hairs and control polar growth. EMBO J. 20, 2779–2788. 11387211 10.1093/emboj/20.11.2779 PMC125484

[jmi12847-bib-0053] Müller, A. , Guan, C. , Gälweiler, L. *et al* (1998) AtPIN2 defines a locus of *Arabidopsis* for root gravitropism control. EMBO J. 17, 6903–6911. 9843496 10.1093/emboj/17.23.6903 PMC1171038

[jmi12847-bib-0054] Nakamura, M. & Grebe, M. (2018) Outer, inner and planar polarity in the *Arabidopsis* root. Curr. Opin. Plant Biol. 41, 46–53. 28869926 10.1016/j.pbi.2017.08.002

[jmi12847-bib-0055] Nakamura, M. & Hashimoto, T. (2009) A mutation in the *Arabidopsis* gamma‐tubulin‐containing complex causes helical growth and abnormal microtubule branching. J. Cell Sci. 122, 2208–2217. 19509058 10.1242/jcs.044131

[jmi12847-bib-0056] Oda, Y. , Otani, T. , Ikenouchi, J. & Furuse, M. (2014) Tricellulin regulates junctional tension of epithelial cells at tricellular contacts through Cdc42. J. Cell Sci. 127, 4201–4212. 25097232 10.1242/jcs.150607

[jmi12847-bib-0057] Panteris, E. , Apostolakos, P. & Galatis, B. (1993a). Microtubule organization, mesophyll cell morphogenesis, and intercellular space formation in *Adiantum capillus‐veneris* leaflets. Protoplasma 172, 97–110. 10.1111/j.1469-8137.1993.tb03899.x 33874586

[jmi12847-bib-0058] Panteris, E. , Apostolakos, P. & Galatis, B. (1993b) Microtubule organization and cell morphogenesis in two semi‐lobed cell types of *Adiantum capillus‐veneris* L. leaflets. New Phytol. 125, 509–520. 10.1111/j.1469-8137.1993.tb03899.x 33874586

[jmi12847-bib-0059] Panteris, E. , Galatis, B. & Apostolakos, P. (1991) Patterns of cortical and perinuclear microtubule organization in meristematic root cells of *Adiantum capillus‐veneris* . Protoplasma 165, 173–188.

[jmi12847-bib-0060] Parker, C.C. , Parker, M.L. , Smith, A.C. & Waldron, K.W. (2001) Pectin distribution at the surface of potato parenchyma cells in relation to cell‐cell adhesion. J. Agric. Food Chem. 49, 4364–4371. 11559139 10.1021/jf0104228

[jmi12847-bib-0061] Parker, C.C. , Parker, M.L. , Smith, A.C. & Waldron, K.W. (2003) Thermal stability of texture in Chinese water chestnut may be dependent on 8,8’‐diferulic acid (aryltetralyn form). *J* Agric. Food Chem. 51, 2034–2039. 10.1021/jf020759p 12643670

[jmi12847-bib-0062] Parr, A.J. , Waldron, K.W. , Ng, A. & Parker, M.L. (1996) The wall‐bound phenolics of Chinese water chestnut (*Eleocharis dulcis*). J. Sci. Food Agric. 71, 501–507.

[jmi12847-bib-0063] Peaucelle, A. , Wightman, R. & Höfte, H. (2015) The control of growth symmetry breaking in the *Arabidopsis* hypocotyl. Curr. Biol. 25, 1746–1752. 26073136 10.1016/j.cub.2015.05.022

[jmi12847-bib-0064] Pillitteri, L.J. , Peterson, K.M. , Horst, R.J. & Torii, K.U. (2011) Molecular profiling of stomatal meristemoids reveals new component of asymmetric cell division and commonalities among stem cell populations in *Arabidopsis* . Plant Cell 23, 3260–3275. 21963668 10.1105/tpc.111.088583 PMC3203429

[jmi12847-bib-0065] Reinhardt, D. , Pesce, E.‐R. , Stieger, P. *et al* 2003 Regulation of phyllotaxis by polar auxin transport. Nature 426, 255–260. 14628043 10.1038/nature02081

[jmi12847-bib-0066] Robert, H.S. , Grunewald, W. , Sauer, M. *et al* (2015) Plant embryogenesis requires AUX/LAX‐mediated auxin influx. Development 142, 702–711. 25617434 10.1242/dev.115832

[jmi12847-bib-0067] Routier‐Kierzkowska, A.‐L. , Weber, A. , Kochova, P. , Felekis, D. , Nelson, B.J. , Kuhlemeier, C. & Smith, R.S. (2012) Cellular force microscopy for *in vivo* measurements of plant tissue mechanics. Plant Physiol. 158, 1514–1522. 22353572 10.1104/pp.111.191460 PMC3343728

[jmi12847-bib-0068] Ruzicka, K. , Strader, L.C. , Bailly, A. *et al* (2010) *Arabidopsis* PIS1 encodes the ABCG37 transporter of auxinic compounds including the auxin precursor indole‐3‐butyric acid. Proc. Nat. Acad. Sci. U.S.A. 107, 10749–10753. 10.1073/pnas.1005878107 PMC2890796 20498067

[jmi12847-bib-0069] Sachs, J. (1877) Über die Anordnung der Zellen in jüngsten Pflanzentheilen. Staehl'sche Buch‐ & Kunsthandlung, Würzburg, Germany.

[jmi12847-bib-0070] Samalova, M. , Mélida, H. , Vilaplana, F. , Bulone, V. , Soanes, D.M. , Talbot, N.J. & Gurr, S.J. (2017) The β208;**1**,3‐glucanosyltransferases (Gels) affect the structure of the rice blast fungal cell wall during appressorium‐mediated plant infection. Cellular Microbiol. 19, e12659. 10.1111/cmi.12659 PMC5396357 27568483

[jmi12847-bib-0071] Staehelin, L.A. (1973) Further observations on the fine structure of freeze‐cleaved tight junctions. J. Cell Sci. 13, 763–786. 4203962 10.1242/jcs.13.3.763

[jmi12847-bib-0072] Strader, L.C. & Bartel, B. (2009) The *Arabidopsis* pleiotropic drug resistance8/ABCG36 ATP binding cassette transporter modulates sensitivity to the auxin precursor indole‐3‐butyric acid. Plant Cell 21, 1992–2007. 19648296 10.1105/tpc.109.065821 PMC2729616

[jmi12847-bib-0073] Suslov, D. , Verbelen, J.‐P. & Vissenberg, K. (2009) Onion epidermis as a new model to study the control of growth anisotropy in higher plants. J. Exp. Bot. 60, 4175–4187. 19684107 10.1093/jxb/erp251

[jmi12847-bib-0074] Swarup, K. , Benková, E. , Swarup, R. *et al* (2008) The auxin influx carrier LAX3 promotes lateral root emergence. Nat. Cell Biol. 10, 946–954. 18622388 10.1038/ncb1754

[jmi12847-bib-0075] Swarup, R. , Friml, J. , Marchant, A. , Ljung, K. , Sandberg, G. , Palme, K. & Bennett, M. (2001) Localization of the auxin permease AUX1 suggests two functionally distinct hormone transport pathways operate in the *Arabidopsis* root apex. Genes Dev. 15, 2648–2653. 11641271 10.1101/gad.210501 PMC312818

[jmi12847-bib-0076] Swarup, R. , Kargul, J. , Marchant, A. *et al* (2004) Structure‐function analysis of the presumptive *Arabidopsis* auxin permease AUX1. Plant Cell 16, 3069–3083. 15486104 10.1105/tpc.104.024737 PMC527199

[jmi12847-bib-0077] Szymańska‐Chargot, M. , Chylińska, M. , Pieczywek, P.M. , Rösch, P. , Schmitt, M. , Popp, J. & Zdunek, A. (2016) Raman imaging of changes in the polysaccharides distribution in the cell wall during apple fruit development and senescence. Planta 243, 935–945. 26733465 10.1007/s00425-015-2456-4 PMC4819746

[jmi12847-bib-0078] Takano, J. , Tanaka, M. , Toyoda, A. *et al* (2010) Polar localization and degradation of *Arabidopsis* boron transporters through distinct trafficking pathways. Proc. Nat. Acad. Sci. U.S.A. 107, 5220–5225. 10.1073/pnas.0910744107 PMC2841934 20194745

[jmi12847-bib-0079] Tirumalai, V.C. , Agarwal, U.P. & Obst, J.R. (1996) Heterogeneity of lignin concentration in cell corner middle lamella of white birch and black spruce. Wood Sci. Technol. 30, 99–104.

[jmi12847-bib-0080] Ugartechea‐Chirino, Y. , Swarup, R. , Swarup, K. , Péret, B. , Whitworth, M. , Bennett, M. & Bougourd, S. (2010) The AUX1 LAX family of auxin influx carriers is required for the establishment of embryonic root cell organization in *Arabidopsis thaliana* . Ann. Bot. 105, 277–289. 19952011 10.1093/aob/mcp287 PMC2814760

[jmi12847-bib-0081] Vaddepalli, P. , Fulton, L. , Wieland, J. , Wassmer, K. , Schaeffer, M. , Ranf, S. & Schneitz, K. (2017) The cell wall‐localized atypical beta‐1,3 glucanase ZERZAUST controls tissue morphogenesis in *Arabidopsis thaliana* . Development 144, 2259–2269. 28507000 10.1242/dev.152231

[jmi12847-bib-0082] Vaughn, K.C. , Bowling, A.J. & Ruel, K.J. (2011) The mechanism for explosive seed dispersal in *Cardamine hirsuta* (Brassicaceae). Am. J. Bot. 98, 1276–1285. 21795731 10.3732/ajb.1000374

[jmi12847-bib-0083] Voigt, B. , Timmers, A.C.J. , Šamaj, J. , Müller, J. , Baluška, F. & Menzel, D. (2005) GFP‐FABD2 fusion construct allows *in vivo* visualization of the dynamic actin cytoskeleton in all cells of *Arabidopsis* seedlings. Euro. J. Cell Biol. 84, 595–608. 10.1016/j.ejcb.2004.11.011 16032928

[jmi12847-bib-0084] Waldron, K.W. , Smith, A.C. , Parr, A.J. , Ng, A. & Parker, M.L. (1997) New approaches to understanding and controlling cell separation in relation to fruit and vegetable texture. Trends Food Sci. Technol. 8, 213–221.

[jmi12847-bib-0085] Wang, P. , Richardson, C. , Hawes, C. & Hussey, P.J. (2016) Arabidopsis NAP1 regulates the formation of autophagosomes. Curr. Biol. 26, 2060–2069. 27451899 10.1016/j.cub.2016.06.008

[jmi12847-bib-0086] Wang, Y.‐S. , Motes, C.M. , Mohamalawari, D.R. & Blancaflor, E.B. (2004) Green fluorescent protein fusions to *Arabidopsis* fimbrin 1 for spatio‐temporal imaging of F‐actin dynamics in roots. Cell Motility Cytoskel. 59, 79–93. 15362112 10.1002/cm.20024

[jmi12847-bib-0087] Wang, Y.‐S. , Yoo, C.‐M. & Blancaflor, E.B. (2008) Improved imaging of actin filaments in transgenic *Arabidopsis* plants expressing a green fluorescent protein fusion to the C‐ and N‐termini of the fimbrin actin‐binding domain 2. New Phytol. 177, 525–536. 18028299 10.1111/j.1469-8137.2007.02261.x

[jmi12847-bib-0088] Wolf, S. & Greiner, S. (2012) Growth control by cell wall pectins. Protoplasma 249(Suppl 2), S169–175. 22215232 10.1007/s00709-011-0371-5

[jmi12847-bib-0089] Yi Chou, E. , Schuetz, M. , Hoffmann, N. , Watanabe, Y. , Sibout, R. & Samuels, A.L. (2018) Distribution, mobility, and anchoring of lignin‐related oxidative enzymes in *Arabidopsis* secondary cell walls. J. Exp. Bot. 69, 1849–1859. 29481639 10.1093/jxb/ery067 PMC6018803

[jmi12847-bib-0090] Yoshida, S. , van der Schuren, A. , van Dop, M. *et al* (2019) A SOSEKI‐based coordinate system interprets global polarity cues in *Arabidopsis* . Nat. Plants 5, 160–166. 30737509 10.1038/s41477-019-0363-6 PMC6420093

[jmi12847-bib-0091] Zamil, M.S. , Yi, H. , Haque, M.A. & Puri, V.M. (2013) Characterizing microscale biological samples under tensile loading: stress–strain behavior of cell wall fragment of onion outer epidermis. Am. J. Bot. 100, 1105–1115. 23720433 10.3732/ajb.1200649

[jmi12847-bib-0092] Zamil, M.S. , Yi, H. & Puri, V.M. (2014) Mechanical characterization of outer epidermal middle lamella of onion under tensile loading. Am. J. Bot. 101, 778–787. 24808543 10.3732/ajb.1300416

[jmi12847-bib-0093] Zeng, Y. , Himmel, M.E. & Ding, S.‐Y. (2017) Visualizing chemical functionality in plant cell walls. Biotech. Biofuels 10, 263. 10.1186/s13068-017-0953-3 PMC5708085 29213316

[jmi12847-bib-0094] Zhou, J.‐J. & Luo, J. (2018) The PIN‐FORMED auxin efflux carriers in plants. Int. J. Mol. Sci. 19, 2759. 10.3390/ijms19092759 PMC6164769 30223430

